# Cerebral blood flow measurements with ^15^O-water PET using a non-invasive machine-learning-derived arterial input function

**DOI:** 10.1177/0271678X21991393

**Published:** 2021-02-08

**Authors:** Samuel Kuttner, Kristoffer Knutsen Wickstrøm, Mark Lubberink, Andreas Tolf, Joachim Burman, Rune Sundset, Robert Jenssen, Lieuwe Appel, Jan Axelsson

**Affiliations:** 1 Department of Clinical Medicine, UiT The Arctic University of Norway, Tromsø, Norway; 2 Department of Physics and Technology, UiT The Arctic University of Norway, Tromsø, Norway; 3 The PET Imaging Center, University Hospital of North Norway, Tromsø, Norway; 4 Department of Surgical Sciences, Radiology, Uppsala University, Uppsala, Sweden; 5 Department of Neuroscience, Uppsala University, Uppsala, Sweden; 6 Department of Radiation Sciences, Umeå University, Umeå, Sweden

**Keywords:** Arterial input function, cerebral blood flow, Gaussian processes, kinetic modelling, machine learning, 15O-water PET

## Abstract

Cerebral blood flow (CBF) can be measured with dynamic positron emission tomography (PET) of ^15^O-labeled water by using tracer kinetic modelling. However, for quantification of regional CBF, an arterial input function (AIF), obtained from arterial blood sampling, is required. In this work we evaluated a novel, non-invasive approach for input function prediction based on machine learning (MLIF), against AIF for CBF PET measurements in human subjects.

Twenty-five subjects underwent two 10 min dynamic ^15^O-water brain PET scans with continuous arterial blood sampling, before (baseline) and following acetazolamide medication. Three different image-derived time-activity curves were automatically segmented from the carotid arteries and used as input into a Gaussian process-based AIF prediction model, considering both baseline and acetazolamide scans as training data. The MLIF approach was evaluated by comparing AIF and MLIF curves, as well as whole-brain grey matter CBF values estimated by kinetic modelling derived with either AIF or MLIF.

The results showed that AIF and MLIF curves were similar and that corresponding CBF values were highly correlated and successfully differentiated before and after acetazolamide medication. In conclusion, our non-invasive MLIF method shows potential to replace the AIF obtained from blood sampling for CBF measurements using ^15^O-water PET and kinetic modelling.

## Introduction

Measurements of cerebral blood flow (CBF) can be used to separate pathological and healthy brain tissue as well as for functional brain research. Tracer kinetic modelling following dynamic ^15^O-water positron emission tomography (PET) imaging with arterial blood sampling is considered the reference standard for CBF measurements.^1–6^ However, arterial cannulation is an invasive, laborious and time-consuming procedure, and may, due to induction of pain and risk for complications, discourage patients and volunteers from participating in research studies. Furthermore, a useful arterial input function (AIF) curve cannot be obtained without careful cross calibration of the blood measurement detector and the PET scanner. In addition, because the blood is most commonly sampled from the radial artery, additional corrections for dispersion and delay of the tracer must be applied, to obtain the true AIF for the brain.^3^,^7–9^

The use of an image-derived input function (IDIF) has been proposed as an alternative approach to overcome the challenges with the AIF.^10^ In brain PET imaging, an IDIF can be measured inside a suitable intracranial blood vessel directly in the reconstructed PET images, for instance in the intracranial carotid arteries.^11–13^ Due to the limited spatial resolution of the PET system, and the need for short time-frames during the first pass of the bolus, image-derived methods suffer from both partial volume effects and image noise. These limitations require complex and standardized methods for partial volume correction and artery delineation, which may be difficult to achieve in practice. Recently, a few clinical studies have suggested the use of integrated PET/magnetic resonance imaging (MRI) for deriving an IDIF in brain, where the latter modality is used for artery delineation or even motion correction.^14–18^ However these methods are sensitive to registration errors between the modalities and require detailed knowledge of the scanner resolution. Another recent study overcame potential misregistration problems and formed a corrected IDIF by deriving total number of counts and artery volume from the two modalities separately.^19^ However, this method was not yet validated with arterial blood sampling and as hybrid PET/MRI is still an emerging modality, to date, image-derived methods are rarely used in larger clinical or research studies.^10^,^20^

Alternatively, a standardized, population-based AIF can be calculated as an average AIF from a group of subjects acquired with the same tracer, injection protocol and population, and scaled to the specific subject.^21^,^22^ However, this method requires at least one blood sample for curve scaling while individual physiological differences and scan-dependent variations are neglected.

An approach on image data with simultaneous estimation of AIF and kinetic parameters has also been reported.^23–25^ This method, however, assumes a known mathematical AIF model and requires at least one late blood sample for parameter estimation. Recently, non-invasive simultaneous estimation methods were developed that obviate the need for the single late blood sample by using additional input variables from electronic health records into the models.^26^,^27^ The limitation of such an approach is that a large set of clinical variables must be collected and handled for each patient. These variables may not necessarily be available in the health records for all patients and may even complicate inclusion of healthy volunteers in research studies.

In this study we use a machine learning-based approach for AIF estimation. Machine learning-methods are especially useful for function estimation and regression.^28^ Briefly, one seeks to determine a function, f, that predicts the machine-learning-derived input function (MLIF), based on an input vector, x, composed of multiple image-derived tissue curves, such that MLIF=f(x). The function f is determined by optimizing hidden parameters to find the best mapping, A$\mathrm{IF}=f\left(x\right),$ for a set of training data, where both the AIF and the tissue curves are known. Once the model has been trained and f is known, the MLIF can be predicted for unseen test data using only the tissue-curves extracted from the image data.^28^

In our previous work, we developed and validated a machine-learning-based input function for ^18^F-fluorodeoxyglucose (FDG) in a mouse PET cohort.^29^ In short, two learning models were evaluated that predicted an AIF from time-activity curves of up to 7 different tissue regions as input. The main limitation with our previous study was the lack of an AIF, thus the models could only be validated against a reference IDIF. However, in mouse PET scanning, the entire body of the mouse fits in the PET field-of-view, thus, time-activity curves from all organs are readily available as input data for the models. We showed that, for instance, the myocardium and liver were important for AIF prediction, because their time-activity curves closely resembled the reference IDIF. In contrast, these blood-rich organs are outside the field-of-view in clinical brain PET imaging, and thus, alternative input curves had to be derived for the MLIF model in the current study.

In the present study, we have further developed the MLIF approach for human ^15^O-water brain PET data and evaluated the models against an AIF obtained from continuous arterial-blood sampling. We aimed to show that an AIF could be accurately predicted by an MLIF model using multiple image-derived input data from the carotid arteries. We hypothesized that there were no significant differences in estimated CBF when using either AIF or MLIF, and investigated similarities and differences between image-derived sampling in the brain versus arterial-sampling from the arm. Further, we investigated whether the MLIF method was capable to predict a clinically relevant CBF difference between scans before and after acetazolamide medication.

## Materials and methods

### Subjects

Pseudonymized data from 25 subjects were retrospectively collected from a completed clinical research study at Uppsala University Hospital. The data comprised both patients with multiple sclerosis (MS) and healthy volunteers (mean age (range) in years: 40 (23–56); F:M 15:10). In this methodological study, we did not differentiate between the two groups as we considered that the subject’s health status had no impact on our evaluation of the MLIF model. Therefore, all authors were blinded for the health status of each subject and thus, no comparisons were made between healthy subjects and MS-patients. The results of the parent study will be reported elsewhere.

The parent study was approved by the Swedish Ethical Review Authority (reference 2014/453). All subjects signed written informed consent prior to inclusion. Since the present work was purely an image analysis methodology study using pseudonymized data, it was not covered by the Swedish or Norwegian regulations on medical research in humans and as such, no additional ethics approval was necessary.

### Image acquisition

All subjects underwent two 10 min dynamic brain PET scans on either an ECAT Exact HR+ stand-alone PET scanner (Siemens, Knoxville, TN; n = 9) or a Discovery MI PET-Computed tomography (CT) scanner (GE Healthcare, Waukesha, MI; n = 16). The scans started simultaneously with an automated bolus injection of 5 MBq/kg ^15^O-water (^15^O-water in 5 ml saline at 1 ml/s followed by 35 ml saline at 2 ml/s). Each subject underwent one scan at baseline and one scan 15–30 min after intravenous administration of acetazolamide (9 mg/kg up to a maximum of 1000 mg; 5 min infusion) such that every subject was its own control. Acetazolamide medication dilates the vascular system and thereby it increases the cerebral arterial blood flow velocity.^30–32^ Attenuation correction was based on a 10 min transmission scan with rotating ^68^Ge rod sources (ECAT) or an ultra-low-dose CT scan (Discovery MI). Images were reconstructed into 26 time-frames (1 × 10, 8 × 5, 4 × 10, 2 × 15, 3 × 20, 2 × 30 and 6 × 60 s). Image reconstruction algorithms were chosen to result in a matching image resolution for the two scanners: ordered subsets expectation maximization with 6 iterations, 8 subsets and a 4 mm Hanning filter (ECAT) and 3 iterations, 16 subsets and a 5 mm Hanning filter (Discovery MI).

In addition, all subjects underwent MRI on a 3 T MRI scanner (Achieva, Philips Healthcare, Best, The Netherlands) with a 32-channel head coil. A three-dimensional T1-weighted gradient echo sequence was obtained with voxel size 1.0 × 1.0 × 1.0 mm^3^, repetition time = 8.2 ms and echo time = 3.7 ms.

### Blood sampling

Continuous arterial blood sampling was performed during 10 min for each scan (3 ml/min) using either an ABSS V3 (Allogg, Mariefred, Sweden; subjects scanned on ECAT) or PBS-100 (Veenstra-Comecer, Joure, The Netherlands; subjects scanned on Discovery MI). A single arterial blood sample was taken through a three-way-valve on the arterial line 5 min post injection and measured in a cross-calibrated well counter for calibration of the continuous arterial blood data.

The measured blood signal, *g*(*t*), was affected by dispersion in the vessels and in the detector system tubes. This could be modelled as a convolution of the true AIF, *C_A_*(*t*), and a dispersion function, *d*(*t*)^8^

(1) $$g\left(t\right)=C_{A}(t)⊗d(t)$$

A mono-exponential dispersion model was assumed^8^

(2) $$d\left(t\right)=\frac{1}{\tau }e^{-\frac{t}{\tau }}$$

where *τ* is the dispersion constant. An expression for the true AIF, *C_A_*(*t*), could be obtained by the Laplace transform^33^

(3) $$C_{A}\left(t\right)=g\left(t\right)+\;\tau \frac{dg}{dt}$$

The dispersion constant was fixed to 15 s for all subjects in this study, as suggested in the literature.^8^

The dispersion-corrected AIFs were delay-corrected, as described in the ‘Image processing’ Section.

A visual assessment of the AIF curves was performed to identify abnormal AIFs due to failures in tracer administration or continuous arterial blood sampling. Three subjects were excluded after the visual assessments.

### Image processing

**Figure 1.**
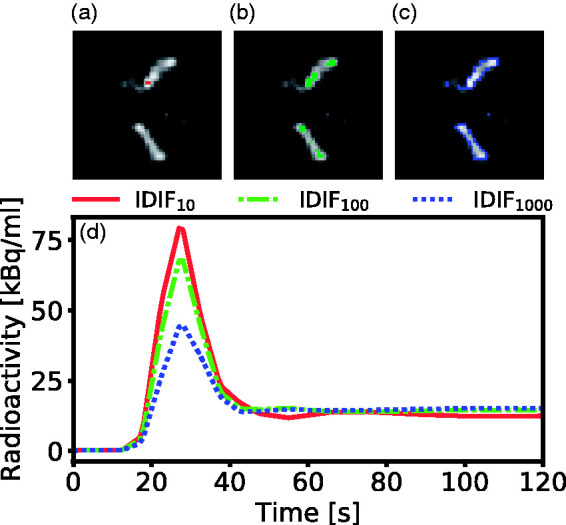
Outline of the VOI thresholding method implemented in this work. (a) to (c) shows an enlarged axial brain PET slice of the optimal time-frame for a representative subject. Highlighted are the parts of the IDIF_10_ (a), IDIF_100_ (b) and IDIF_1000_ (c) comprising the 10, 100 and 1000 highest intensity voxels. (d) The resulting time-activity curves during the first two minutes for IDIF_10-1000_. The IDIF_10_ captures the highest activity in the carotid artery, while the IDIF_100_ and IDIF_1000_ shows a lower activity due to a higher number of voxels included.

All images were corrected for inter-frame motion using in-house written software in Matlab (Mathworks, MA, USA). A simple and objective multi-VOI thresholding method, that could capture blood information from the carotid arteries, was empirically developed. First, to remove noise close to the edge slices, a PET search-volume was defined by trimming 20 voxels in the x-y-plane periphery, and by removing 5 slices in the z-direction. The algorithm for identifying the time-frame for carotid-VOI thresholding was based on a frame-wise graph of whole-brain gray-matter total intensity. The time-frame used for VOI thresholding was the first frame where the total intensity was larger than 25% of the maximum value in this graph. Three VOIs were generated, comprising the 10, 100 and 1000 highest-intensity voxels (Figure 1a to c). The median voxel value was derived for each time-frame and VOI, resulting in three IDIF time-activity curves, named IDIF_10_, IDIF_100_ and IDIF_1000_ (Figure 1d), which could be interpreted as three different image-derived blood-curves with different amounts of partial volume effect.

Subsequently, to match the AIF sampling, all PET data were interpolated linearly to one second time framing. To correct for delay between the AIF and the PET data, the dispersion corrected AIF was shifted to provide the best overlap with the IDIF_10_ curve, i.e. where the dot product between the two curves was maximized.

In all following analyses, only PET and AIF data from 0–6 min were used, to minimize noise from late parts of the scans. Calibration of the AIF curves with the single arterial blood sample was only conducted for the training data, and thus no AIF or blood sample was required for the test data.

To extract time-activity curves for whole-brain grey matter, T1-weighted MRI images were co-registered to PET images and segmented using SPM8 (The Wellcome Centre for Human Neuroimaging, UCL Queen Square Institute of Neurology, London, UK). All image analysis was performed in the native PET image space.

### Function prediction using Gaussian processes

Gaussian processes (GP) is a well-known, non-parametric Bayesian regression method which has been frequently used within machine learning for data-driven function estimation and regression tasks.^34,35^ One advantage with GP is that it predicts not only the mean function, but also its variance, thus providing an uncertainty measure of the model.^36^ Furthermore, GP, is known to work well with sparse training datasets, as opposed to neural networks.^37,38^

In GP regression, the output *y*, is approximated by a probability distribution of functions of the input, x, such that $f(x)∼GP\left(m\left(x\right),\;k\left(x,x\prime \right)\right)$, which is a generalization of the multivariate Gaussian distribution to infinitely many variables. Here, $m\left(x\right)$ is a mean function and $k\left(x,x\prime \right)$ is a covariance function.^36^ Assuming *N* available input-output training samples in a dataset ${\{x}_{n},y_{n}{\}}_{n=1}^{N}$, each including the three IDIF time-activity curves,$\;x_{n}$ (see Figure 1), with corresponding known AIF,$\;y_{n}$. Then the mean value MLIF of the test sample, $E\left[y_{*}\right]$, and the variance, $V\left[y_{*}\right]$, can be predicted by

(4) $$E^{[y_{*}}]=k_{*}^{T}(K+\sigma_{\epsilon }^{2}I{)}^{-1}y$$

(5) $$V{[y}_{*}]=k\left(x_{*},x_{*}\right)-k_{*}^{T}(K+\sigma_{\epsilon }^{2}I{)}^{-1}k_{*}$$

Here $k_{*}$ is the covariance between the training samples $x_{n}\;$and the test sample $x_{*}$; $K=k\left(x_{n},x_{m}\right)$ is the covariance between all training samples;$\;\sigma_{\epsilon }^{2}I$ is a scalar matrix with diagonal elements equal to the noise level; $k\left(x_{*},x_{*}\right)$ is the covariance between the test sample and itself.^36^

### Input function prediction

For input function prediction, leave-one-out cross validation was employed, which is a common validation method in machine learning with limited amounts of data.^28^ In short, one sample was withdrawn from the dataset and assigned as test sample, while the remaining samples were allocated for training. The three time-activity curves (Figure 1) were used as input vectors into the GP framework, and the MLIF and variance of the test sample were predicted using equations (4) and (5), respectively. The process was repeated by assigning a new sample as test sample, until all samples had been tested once.

In all experiments, the Matérn covariance function was chosen, with *ν* = 5*/*2, because it has been reported to generate smooth functions.^36^ Data normalization was applied on the input-IDIFs, which is a well-known approach to improve convergence of machine learning models.^39^ Normalization of the IDIF_10_ curves was performed by identifying the IDIF_10_ curve with the highest peak value among all subjects. Subsequently, each IDIF_10_ curve was normalized by dividing with this peak value. Similarly, the IDIF_100_ and IDIF_1000_ curves were normalized independently with their respectively found highest peak value among the subjects. Thus, the model was trained with three different normalized IDIFs with values ranging between 0 and 1. The normalization was only a scale factor, meaning that relative amplitudes between subjects remained. GP regression was implemented in Python 3.6.8, using GPflow 1.2.0, in which the matrix inversion of equation (4) was approximated by Cholesky decomposition. The hyperparameters of the covariance function were optimized by maximizing the logarithm of the marginal likelihood of the training data.^40^

### Kinetic modelling

Quantification of CBF was performed on the whole-brain grey-matter region. A single-tissue compartment model was used to generate CBF values. This model assumes that water can diffuse freely between vascular and tissue space, with activity concentrations *C_A_* and *C_T,_* respectively, and is described by the following equation^41,42^

(6) $$\frac{dC_{T}(t)}{dt}={K_{1}\cdot C}_{A}\left(t\right)-{k_{2}\cdot C}_{T}(t)$$

where *C_A_*(*t*) is the whole-blood arterial time-activity curve, also known as the AIF. The solution to equation (6) is given by^43^

(7) $$C_{T}(t)={K_{1}\cdot C}_{A}\left(t\right)⊗e^{-k_{2}\cdot t}$$

where ⊗ denotes mathematical convolution. The activity concentration measured with PET, *C_PET_*, is modelled as the sum of the tissue compartment, *C_T_* (*t*), and the fractional arterial blood volume in the tissue, V_A_, such that

(8) $$C_{\mathrm{PET}}\left(t\right)=\left(1-V_{A}\right)\cdot C_{T}\left(t\right)+V_{A}{\cdot C}_{A}(t)$$

For tracers with high extraction fraction relative to the blood flow, such as ^15^O-water, CBF equals *K*_1_.^43,44^

### Evaluation design

The aim of the current work was to investigate whether an AIF could be accurately predicted using an MLIF model in baseline and acetazolamide scans of the same subject. In a first case, a GP model named MLIF_1_ was trained and subsequently tested on baseline scans using leave-one-out cross validation. Similarly, another GP model, named MLIF_2_, was trained and subsequently tested on acetazolamide scans using leave-one-out cross validation.

In a routine setting, it is of interest to train a predictive model on normal subjects and apply the same model on a disease, or medicated group. Therefore, in a second case, an additional GP model named MLIF^*^_1_, was trained on all baseline scans, and subsequently tested on all acetazolamide scans. Here, the asterisk (*) in MLIF^*^_1_ emphasises that all subjects from the baseline scan were included in the training data for this model, as opposed to the leave-one-out training for MLIF_1_. Essentially, one might see MLIF^*^_1_ and MLIF_1_ as two models trained on the same dataset, as the difference is only one subject.

Finally, we hypothesized that MLIF^*^_1_ might be more representative for a local AIF in the brain, compared to an AIF sampled in the wrist. Therefore, in a third case, we aimed to evaluate the CBF increase from baseline to acetazolamide scans obtained by the two different methods in *case 1* and *case 2*, by using the carotid arteries with the ten highest-intensity voxels (IDF_10_) as input function during kinetic modelling.

The evaluation design is summarized in Table 1.

**Table 1.** The evaluation design of the MLIF method.

Case	Training data	Testing data	Input function	Procedure
1	Baseline	Baseline	MLIF_1_	Leave-one-out
	Acetazolamide	Acetazolamide	MLIF_2_	Leave-one-out
2	Baseline	Baseline	MLIF_1_	Leave-one-out
	Baseline	Acetazolamide	MLIF_1_*	All baseline scans
3	^a^	Baseline	IDIF_10_^a^	Only used to calculated change between baseline and acetazolamide CBF
	^a^	Acetazolamide	IDIF_10_^a^	

^a^In *case 3*, no GP prediction was used, but instead, kinetic modelling was based on the 10 highest-intensity carotid artery voxels (IDIF_10_) as input function.

### Evaluation methods

The GP predicted MLIF curves were first compared point-by-point to AIF using orthogonal regression. Subject scans with regression slopes that were more than three scaled-median absolute-deviations away from the median slope, were considered outliers, and removed from further model comparisons.^45^ Time–activity curves averaged over subjects were calculated for: whole-brain grey matter, AIF, the MLIF models and IDIF_10_. CBF and V_A_ were estimated for whole-brain grey matter using kinetic modelling with both AIF and the GP-predicted MLIF (*case 1* and *case 2*) as well as with the IDIF_10_ time-activity curve as input function (*case 3*).

MLIF-based CBF estimates were compared with the one based on AIF by paired t-test (*α* = 0.05), ratio calculation, orthogonal regression and Bland-Altman plots.^46^ Normality was assessed using quantile–quantile plots.

The GP variance (equation (5)) was considered as a measure of prediction error. This measure was evaluated by the relationship between the magnitude of the CBF-ratio (CBF_MLIF_/CBF_AIF_) and GP variance for *case 1*.

## Results

CBF values based on AIF (CBF_AIF_) and MLIF (CBF_MLIF_) are shown in Supplementary Table S1. The average CBF_AIF_ and CBF_MLIF_ were similar for both baseline and acetazolamide scans, about 0.45 and 0.60 ml·min^−1^·g^−1^, respectively. The mean CBF_MLIF_/CBF_AIF_ ratio was 1.04 and 1.03 for baseline and acetazolamide, respectively. No significant differences were found between the average CBF_AIF_ and CBF_MLIF_ for either scan.

**Figure 2.**
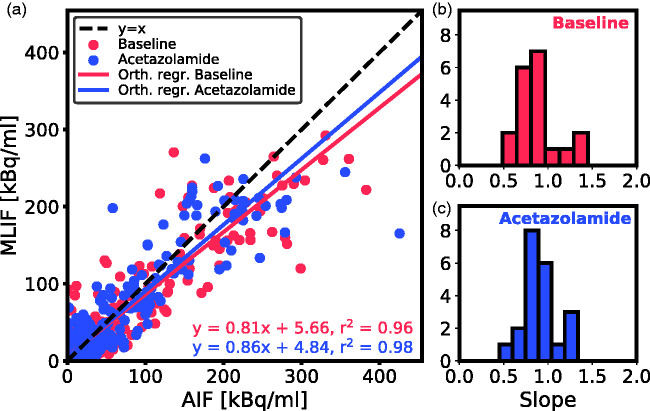
(a) Scatter plot of MLIF and AIF data points for all subjects for baseline (blue) and acetazolamide (red) scans based on *case 1*. The 1 s time frames were interpolated back to the original PET time framing (26 time-frames). The solid lines are the orthogonal regression fits. (b and c) Histogram of orthogonal regression slopes for individual subjects for baseline (b) and acetazolamide (c) scans.

Individual data points for MLIF and AIF from *case 1* are shown as a scatter plot in Figure 2(a). Based on the outlier removal criterion, four scans were removed from model comparisons. There is a strong overall linear relationship between AIF and MLIF curves for both baseline (slope: 0.8) and acetazolamide scans (slope: 0.9). Individual r^2^ values were high for both baseline (mean: 0.90, SD: 0.1) and acetazolamide (mean: 0.93, SD: 0.07). Histograms of slope values indicate slopes close to unity for most subjects for both baseline (median slope: 0.86, Figure 2b) and acetazolamide (median slope: 0.87, Figure 2c).

**Figure 3.**
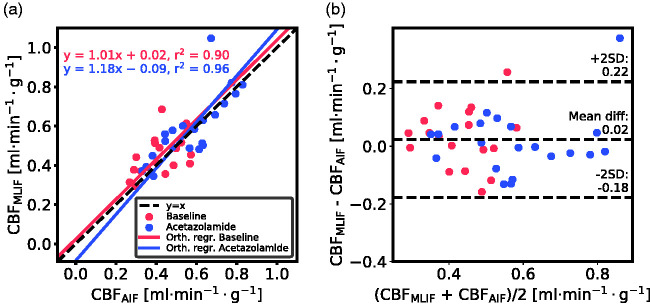
Evaluation of the GP-predicted MLIF for baseline (blue) and acetazolamide (red) scans based on *case 1*. (a) Scatter plot of MLIF-based and AIF-based CBF. The solid lines are the orthogonal regression fits. (b) Bland-Altman plot of *case 1*.

Displaying the CBF data from *case 1* as a scatter plot (Figure 3a), a strong linear relationship and high overall correlation (r^2^>0.9) between CBF_AIF_ and CBF_MLIF_ was obtained. Bland-Altman analysis (Figure 3b) exhibited a prediction bias close to zero.

The fractional arterial blood volume, V_A_, from equation (8), was found to be near zero for all scans (0.001 ± 0.003).

**Figure 4.**
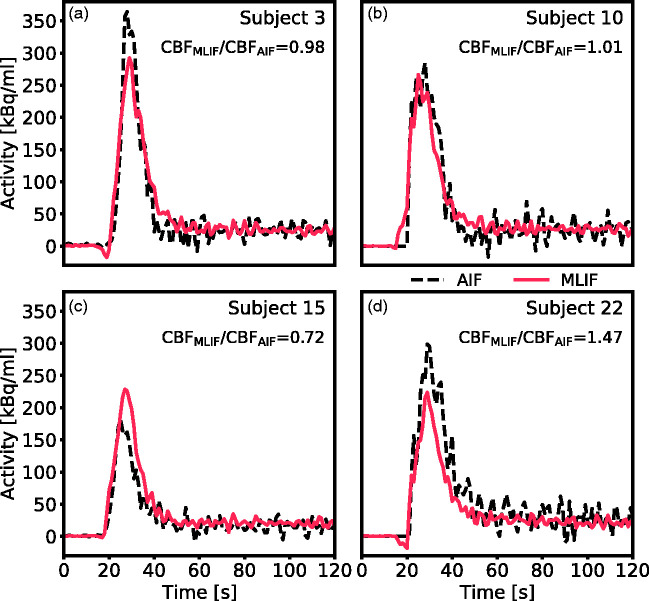
Comparison of AIF (black dashed line) and GP-predicted MLIF (red line) for the baseline scan of four representative subjects. (a and b) Two scans with less than 3% difference between CBF_AIF_ and CBF_MLIF_. (c) Representative example of a scan where the MLIF overpredicts the AIF peak, resulting in an underestimation of the calculated CBF. (d) Representative example of a scan where the MLIF underpredicts the AIF peak, resulting in an overestimation of the calculated CBF.

As a visual illustration of the effect of prediction errors on estimation of CBF, the AIF and GP-predicted MLIF from the baseline scan for four representative subjects are shown in Figure 4. Figure 4(a) and (b) comprises two examples with less than 3% difference between CBF_AIF_ and CBF_MLIF_, while Figure 4(c) and (d) display cases with substantial differences between both methods. Based on the CBF ratios (CBF_MLIF_/CBF_AIF_), it can be observed that an overprediction of the AIF peak (Figure 4c) results in an underestimation of CBF, while an underpredicted AIF peak (Figure 4d) ends in an overestimated CBF.

**Figure 5.**
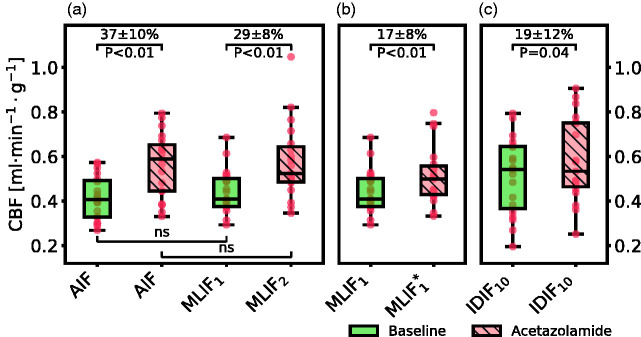
Box plot of estimated whole-brain grey matter CBF for baseline and acetazolamide scans, when using different input functions for *case 1* (a), *case 2* (b) and *case 3* (c). For an explanation of the cases, see Table 1. For visual purposes, the data in panel (c) was scaled to match the range of the AIF-based CBF values in panel (a). Percentage differences are shown as mean ± 95% confidence interval, and P values are based on paired t-test. In the box plots, red points indicate the data points; the horizontal line and the black box represent median and interquartile range (25th to 75th percentile), respectively; the whiskers indicate the maximum and minimum data point up to 1.5 × interquartile range.

Figure 5 displays box plots of whole-brain grey matter CBF for baseline and acetazolamide scans, when using different prediction models for MLIF (see Table 1).

The GP variance was evaluated as prediction error measure in *case 1* (Supplementary Figure S1). No relationship was found between the magnitude of the CBF_MLIF_/CBF_AIF_ ratios and the GP variance values. The predicted variance was not further considered in this work.

In *case 1* we evaluated the MLIF method by training and applying separate GP models for baseline (MLIF_1_) and acetazolamide (MLIF_2_) data. There were no significant differences between mean CBF_AIF_ and mean CBF_MLIF_ in neither baseline, nor acetazolamide scans. Furthermore, following acetazolamide medication, we found on average 37% increase (P *<* 0.01) in mean CBF_AIF_ and 29% increase (P *<* 0.01) in mean CBF_MLIF_ (Figure 5a). For *case 1*, both the AIF and the MLIF based methods resulted in similar CBF values, while the CBF increase after acetazolamide medication was lower for the MLIF method. The correlation coefficient between AIF and MLIF-based CBF changes for *case 1* was 0.62.

In *case 2*, a predictive model (MLIF^*^_1_) was trained on baseline scans and then applied on acetazolamide scans. Similar to MLIF_1_, the average CBF_MLIF*1_ was non-significantly different from the corresponding CBF_AIF_ after acetazolamide medication. Also, there was still a significant increase (17%, P *<* 0.01) in CBF between baseline and acetazolamide scans (Figure 5b), but notably smaller than the 29% increase observed in *case 1.* The correlation coefficient between AIF and MLIF-based CBF changes for *case 2* was 0.14.

In a final case, the relative CBF increase from baseline to acetazolamide scans was investigated by using the IDIF_10_ as input function for each scan. A significant CBF increase of 19% (P = 0.04) was found after acetazolamide medication (Figure 5c), which was comparable to the change observed in *case 2*.

**Figure 6.**
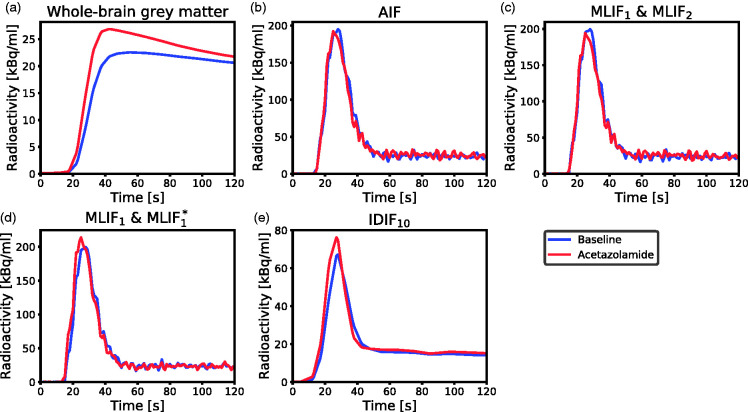
Mean time-activity curves across subjects for baseline (blue) and acetazolamide (red) scans during the first 2 min of PET scanning. (a) Measured radioactivity uptake in whole-brain grey matter. (b) AIF. (c) MLIF_1_ model for baseline, and MLIF_2_ for acetazolamide scans. (d) MLIF_1_ model for baseline, and MLIF^*^_1_ for acetazolamide scans. (e) IDIF_10_ input function based on the 10 highest voxels in the carotid arteries.

As CBF is based on underlying time-activity curves, we proceeded to investigate the differences in CBF after acetazolamide medication in the first two cases by inspecting the mean time-activity curves across subjects in Figure 6. We observed that the local brain input function (IDIF_10_) (Figure 6e) showed a shape-dependence on acetazolamide medication which was not reflected in the AIF measured in the wrist (Figure 6b). In the AIF, the baseline and acetazolamide curves were similar, while for IDIF_10_, the acetazolamide curve had a larger area-under-curve compared to baseline. Also, for IDIF_10_, there was a slight shift in the mean time–activity curves between baseline and acetazolamide, which was not visible for the AIF. Thus, the AIF measured in the wrist does not reflect physiological changes due to acetazolamide, which are apparent in the local brain input (IDIF_10_).

## Discussion

Tracer kinetic modelling of dynamic PET data requires accurate knowledge of an AIF, conventionally measured through arterial blood sampling. Our aim was to investigate whether an AIF could be predicted as accurately by an MLIF model using solely image-derived input data from the carotid arteries.

AIF and predicted MLIF curves were found to be similar, with no significant difference between whole-brain grey matter CBF_AIF_ and CBF_MLIF_ estimates. Furthermore, the correlation between the CBF estimates was r^2^>0.9 and the mean differences were close to zero. The MLIF model was also able to accurately predict an increased CBF after acetazolamide medication, when trained on post-acetazolamide data. Altogether the results indicate that the MLIF method has potential as an alternative AIF for generation of CBF values using ^15^O-water PET and kinetic modelling, which in clinical practice implies evading of arterial cannulation.

Initially, we evaluated an MLIF model trained on pooled baseline and acetazolamide data. However, this resulted in inferior generalization to new samples compared to when training was done separately for baseline and acetazolamide scans (data not shown). The reason for this difference between the approaches may be because the input IDIFs under the two conditions vary in amplitude (Figure 6e), while the AIF (Figure 6b) does not. Therefore, in a first case, input functions of baseline scans were predicted using an MLIF model trained on baseline data (MLIF_1_), and, similarly, input functions of acetazolamide scans were predicted using an MLIF model trained on acetazolamide data (MLIF_2_). There was a strong linear relationship between the data points from AIF and predicted MLIF curves, although the peak values were systematically underestimated (slope <1 in Figure 2). In the time-activity curves, the number of high data values, acquired during the first pass peak, is low compared to the number of low values, acquired during the rest of the scan. Also, the standard deviation around the peaks was observed to be larger than at the tails (data not shown). We speculate that this imbalance affects the GP models and results in the systematically underestimated peak values. Previous work has shown that when data is limited, the GP model may underestimate the mean function.^47^

For both baseline and acetazolamide, the mean time-activity curves for AIF (Figure 6b), and MLIF_1_, MLIF_2_ (Figure 6c) appeared similar in shape. Following kinetic modelling, the average CBF values obtained using an AIF are in line with previously published work before^48,49^ and after administration of acetazolamide.^48^ No significant differences were found between AIF-based and MLIF-based CBF estimates in whole-brain grey matter for neither baseline nor acetazolamide scans (Figure 5a). Also, a slope close to unity, an r^2^>0.9 and low bias between CBF_AIF_ and CBF_MLIF_ estimates pointed towards an acceptable agreement of both methods (Figure 3a). Across both baseline and acetazolamide scans, differences between CBF_AIF_ and CBF_MLIF_ estimates were relatively small (Figure 3b), although several subjects had relative CBF errors of > 20% for the baseline scans while there was a somewhat lower spread for the acetazolamide scans. Our hypothesis is that a GP model trained on acetazolamide data (MLIF_2_) generalize better to new samples, compared to a model trained on baseline data (MLIF_1_). We speculate that the reason for this is that a larger range of CBF values is a result from a larger range of input values (IDIF_10-1000_), for acetazolamide, compared to baseline scans. This may have resulted in MLIF_2_ being valid for a wider range of unseen samples, compared to MLIF_1_, as mentioned and illustrated in Supplementary material. Nevertheless, the CBF increase between baseline and acetazolamide scans was highly correlated to that of the AIF in *case 1*. Furthermore, it was observed that the shape of the input function had an impact on the accuracy of the MLIF-based CBF estimates (Figure 4). Evidently, an underpredicted AIF peak resulted in an overestimated CBF while an overpredicted AIF peak caused an underestimation of CBF. This can be explained by the inverse relationship between K_1_ and C_A_ in equation (7).

In a second case, we trained an MLIF model on all baseline scans and then applied that model on the acetazolamide scans (MLIF^*^_1_). The idea was to resemble a situation where the baseline scans reflected a database comprising healthy subject with a normal CBF whereas the acetazolamide scans reflected clinical data from patients with an altered CBF. Even in this scenario significant differences were found between the whole-brain grey matter CBF from the baseline and acetazolamide scan. However, the CBF increase between baseline and acetazolamide scans in this case displayed small between-subject variation (data not shown) and, maybe because of that, low correlation to the AIF CBF changes. Furthermore, unexpectedly, the difference in CBF was only 17% (Figure 5b) compared to 29% in the previous case (Figure 5a).

We found this difference between baseline and acetazolamide response striking. The local brain input function (IDIF_10_) (Figure 6e) showed a shape-dependence on acetazolamide which was not reflected in the AIF measured in the wrist (Figure 6b). Consequently, CBF calculated with a local brain input would by necessity be different from AIF-based CBF. This effect remained also for the MLIF_1_ and MLIF_1_^*^ models (Figure 6d) and could possibly explain the observed differences in CBF change.

In order to quantify the differences in CBF after acetazolamide medication in the first two cases, we attempted using a carotid artery region (IDIF_10_) as input function for both baseline and acetazolamide scans (*case 3*). In *case 3* a significant difference of 19% was found between baseline and acetazolamide scans which was similar to the observed difference found in *case 2*. This supports that the difference in acetazolamide provocation results were caused by the different input functions in brain (*case 2*, Figure 6d) and in the wrist (*case 1*, Figure 6c). Note that, *case 3* was used only to investigate the relative increase in CBF between baseline and acetazolamide scans found in *case 1* and *case 2*. IDIF_10_ cannot be used as a substitute for AIF, due to the limitations of image derived methods, as described in the Introduction.

We suggest that the above discussed difference in input function curve shape might be explained in part by effects on the vascular system after acetazolamide medication. Acetazolamide dilates the vascular system and increases the cerebral blood flow velocity,^30–32^ which explains the increased mean time–activity curve in whole-brain grey matter after acetazolamide medication (Figure 6a). A lower back-pressure due to dilated vasculature together with increased blood velocity^30,31^ could possibly also explain the observed effect on MLIF^*^_1_ (Figure 6d) and on the IDIF (Figure 6e). Figure 6e also indicated that the ^15^O-water tracer arrived earlier to the brain after acetazolamide medication compared to the baseline scan, resulting in a slight shift of the mean time–activity curves. This observation might also support that the differences in the IDIF_10_ peaks between scans were partly due to an enhanced cerebral blood-flow velocity in the acetazolamide scan. An additional contributing effect to the increased amplitude of the IDIF after acetazolamide may be an increased spill-over from tissue due to increased brain uptake.

In summary, when training and testing on the same scans, similar CBF estimates for whole-brain grey matter are obtained when using AIF and MLIF (*case 1*). However, when using baseline scans for training followed by applying the model to the acetazolamide scans (*case 2*) the blood input curves are higher for the MLIF model, possibly due to the increase in blood flow velocity after acetazolamide medication. Consequently, CBF_MLIF_ for whole-brain grey matter was lower for acetazolamide, compared to baseline, and the difference in CBF before and after acetazolamide medication was reduced from 29% to 17%. Although these relative changes were different for *case 1* and *case 2*, both were significant and hence suggesting that the MLIF method has a clinical potential to differentiate baseline from acetazolamide scans.

A prerequisite for the MLIF approach is that representative training data have been collected for the specific tracer and imaging system, including both images and blood AIFs. Once an MLIF model has been trained, it offers several advantages, compared to various other image-derived and population-based methods.^11–13^,^20–25^ A trained MLIF model is a non-invasive method describing both the shape and the amplitude of an AIF, without any need for calibration blood samples. The MLIF models represents a learned transformation, that directly maps the image-derived input data, to a ready-to-use AIF, by inherently correcting for partial volume effect, with no predefined assumption of the model function. Furthermore, the input data required by the MLIF approach consists of only three carotid artery regions which can be objectively and automatically segmented in the PET images. This makes the MLIF approach simple and convenient to use, without the need for MRI-based artery segmentation.^14–19^

One limitation of this study is that the GP models were trained on at most 22 samples (MLIF^*^_1_), which might have resulted in an inferior generalization of the model to new samples which were dissimilar from the training data. To investigate the robustness of the MLIF model to unseen test data, new IDIF curves were created by scaling the existing input IDIF time–activity curves for each subject during the leave-one-out testing (Supplementary material). When the input data was scaled, the MLIF model was stable for input ranges encountered in the training data. For higher and lower scale factors, the model performance was gradually degraded (Supplementary Figure S2A). The drop of performance of machine learning models outside the range of training data, so called *domain shift*, is expected and well known.^50–52^

In a clinical setting where an existing MLIF model is applied to a patient, it is important that the resulting CBF is reliable. We have reported four outliers based on abnormal regression slopes between the AIF and MLIF data points. However, we cannot know if it was the AIF or the MLIF curves that were abnormal. The corresponding CBF values for these outlier scans all indicate that the AIF was abnormally high as indicated by CBF_MLIF_/CBF_AIF_ ≪ 1 (Table S1). This suggests that the outlier was caused by the AIF and not by the MLIF model. Future research should investigate methods for quality control of predicted MLIF curves and CBF values from a trained model.

Another limitation of the study is the combination of healthy and MS patients in the data set, as well as data from two different scanners. This increased the heterogeneity in the input data to the MLIF models, and in combination with the effects of acetazolamide, this might have affected the results. However, the limited number of subjects does not allow to study these effects further in detail. Further, the evaluation of our MLIF model was mainly based on differences in whole-brain gray matter CBF. We expect similar results to be obtained for CBF in smaller brain regions, but this aspect should be investigated in future studies. Also, the test-retest variability of the MLIF method should be investigated and compared to that of the blood AIF,^53^ as well as the evaluation of the model sensitivity in relation to aspects such as injected dose, time-framing, reconstruction settings and different type of scanners. Finally, the generalizability of the MLIF method to other diseases should be investigated. For example, it is known that carotid stenosis alters the temporal shape of the AIF,^54^ which might have implications for MLIF models trained on baseline scans and applied to patients with pathological arterial vasculature.

In this study, different MLIF models were evaluated with ^15^O-labeled water. In our previous research^29^ a machine learning approach was also feasible for AIF prediction using FDG, although not yet evaluated in clinical data. Thus, we suggest that the method can be adopted to other tracers by merely training similar MLIF models. With proper validation, it may also be conceivable that tracers requiring metabolite correction of the AIF can be included in the prediction model. As for all data-driven models, the accuracy of the MLIF approach for a particular PET application will depend on the quality and quantity of the available training data. Nevertheless, MLIF opens for simplified and non-invasive input function measurements, and thereby potentially eliminating the need for extensive arterial blood sampling in future PET studies.

In conclusion, we demonstrated that our non-invasive MLIF prediction method may be a viable alternative for CBF measurements using ^15^O-water PET and kinetic modelling, which in clinical practice implies evading of arterial cannulation. The MLIF method successfully differentiated CBF values before and after acetazolamide medication.

## Supplemental Material

sj-pdf-1-jcb-10.1177_0271678X21991393 - Supplemental material for Cerebral blood flow measurements with ^15^O-water PET using a non-invasive machine-learning-derived arterial input function Click here for additional data file. Supplemental material, sj-pdf-1-jcb-10.1177_0271678X21991393 for Cerebral blood flow measurements with ^15^O-water PET using a non-invasive machine-learning-derived arterial input function by Samuel Kuttner, Kristoffer Knutsen Wickstrøm, Mark Lubberink, Andreas Tolf, Joachim Burman, Rune Sundset, Robert Jenssen, Lieuwe Appel and Jan Axelsson in Journal of Cerebral Blood Flow & Metabolism
