# Supplementary material for: Cerebral blood flow measurements with 15O-water PET using a non-invasive machine-learning-derived arterial input function
Source: J Cereb Blood Flow Metab. 2021 Feb 8;41(9):2229–41. doi: 10.1177/0271678X21991393 (PMC8392760; doi:10.1177/0271678X21991393)
Supplement: sj-pdf-1-jcb-10.1177_0271678X21991393 - Supplemental material for Cerebral blood flow measurements with 15O-water PET using a non-invasive machine-learning-derived arterial input function [file sj-pdf-1-jcb-10.1177_0271678X21991393.pdf]

Supplementary material

Cerebral blood flow measurements with ^{15}O-water PET using a non-invasive machine-learning-derived arterial input function

Samuel Kuttner[1,2,3*], Kristoffer Knutsen Wickstrøm[2], Mark Lubberink[4], Andreas Tolf[5], Joachim Burman[5], Rune Sundset[1,3], Robert Jenssen[2], Lieuwe Appel[4], Jan Axelsson[6]

[1]Nuclear Medicine and Radiation Biology Research Group, Department of Clinical Medicine, UiT The Arctic University of Norway, Tromsø, Norway.
[2]UiT Machine Learning Group, Department of Physics and Technology, UiT The Arctic University of Norway, Tromsø, Norway.
[3]The PET Imaging Center, University Hospital of North Norway, Tromsø, Norway.
[4]Department of Surgical Sciences, Radiology, Uppsala University, Uppsala, Sweden.
[5]Department of Neuroscience, Uppsala University, Uppsala, Sweden.
[6]Department of Radiation Sciences, Umeå University, Umeå, Sweden.

*Corresponding and first author:
Samuel Kuttner, Nuclear Medicine and Radiation Biology Research Group, Department of Clinical Medicine, UiT The Arctic University of Norway, 9037 Tromsø, Norway
Phone: +47-77 66 99 53
e-mail: samuel.kuttner@uit.no
Twitter: @samuel_kuttner

Table S1. Comparison of individual whole-brain grey matter CBF values for baseline and acetazolamide ^{15}O-water brain PET scans estimated by kinetic modelling using AIF and GP-predicted MLIF as input function. Ratios are expressed as CBF_{MLIF}/CBF_{AIF}. For subjects marked with asterisk (*), the AIF and GP-predicted MLIF from the baseline scan are shown in Figure 2. Scans marked with dagger (†) were outliers according to the slope-based criterion defined in the main text.

	CBF [ml·min^{-1}·g^{-1}]					
	Baseline			Acetazolamide		
Subject	CBF_{AIF}	CBF_{MLIF1}	Ratio	CBF_{AIF}	CBF_{MLIF2}	Ratio
1	0.55	0.61	1.12	0.69	0.66	0.95
2	0.29	0.38	1.30	0.35	0.39	1.12
3*	0.50	0.49	0.98	0.63	0.63	1.00
4	0.33	0.37	1.13	0.48	0.49	1.03
5	0.39	0.51	1.30	0.59	0.59	0.99
6	0.39	0.53	1.34	0.53	0.60	1.13
7	0.30	0.29	0.98	0.33	0.37	1.12
8	0.42	0.49	1.18	0.79	0.77	0.96
9	0.45	0.36	0.80	0.57	0.49	0.86
10*	0.41	0.41	1.01	0.38	0.45	1.17
11	0.36	0.39	1.10	0.45	0.52	1.18
12	0.32	0.37	1.14	0.61	0.48	0.79
13	0.27	0.31	1.17	0.39	0.35	0.89
14	0.43	0.69	1.60	0.67	1.05	1.56
15*	0.57	0.41	0.72	0.63	0.50	0.79
16	0.52	0.52	0.99	0.74	0.71	0.97
17	0.57	0.45	0.79	0.77	0.82	1.06
18	0.76†	0.44†	0.58†	0.83	0.81	0.98
19	0.91†	0.49†	0.54†	0.48	0.58	1.20
20	0.49	0.40	0.82	0.63	0.51	0.82
21	0.57†	0.42†	0.74†	0.69†	0.54†	0.78†
22*	0.30	0.44	1.47	0.44	0.56	1.26
Mean	0.46	0.44	1.04	0.58	0.59	1.03
SD	0.16	0.09	0.28	0.15	0.17	0.19
P	0.67			0.73		

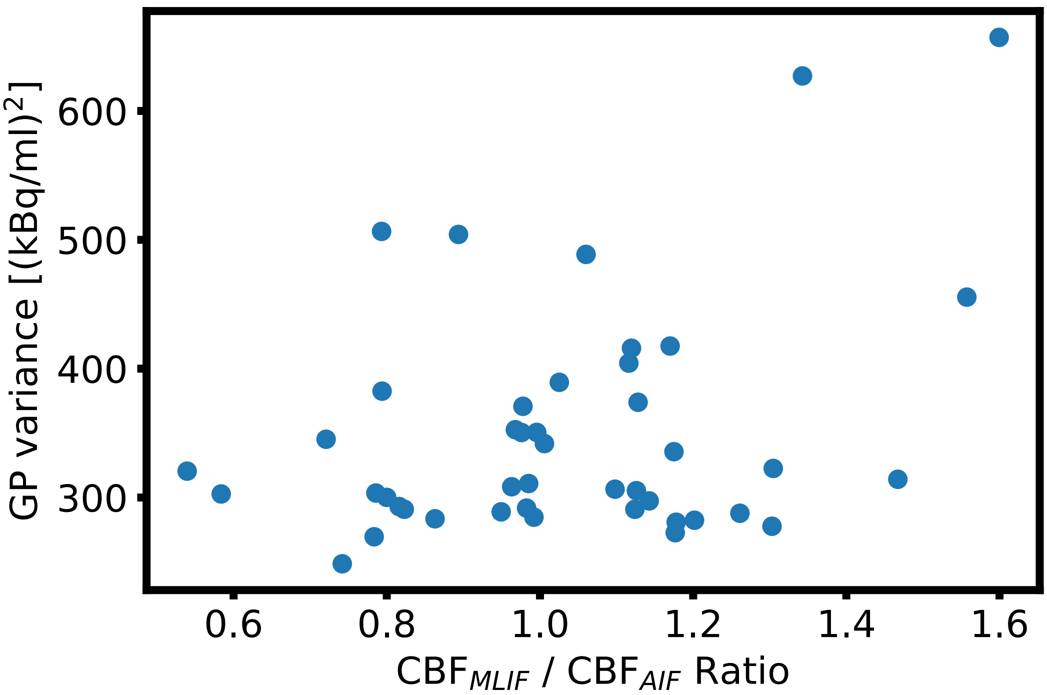

Figure S1. Scatter plot of predicted GP variance versus CBF_{MLIF}/CBF_{AIF} ratio. No clear relation between GP variance and high or low ratio values was observed in the data.

MLIF scale dependency

We have further investigated how the MLIF model responds to input IDIFs outside the range of the training data. This was evaluated by multiplying the input IDIF time-activity curves of the test subject with a scale factor during the leave-one-out testing. Thus, the MLIF model was trained on non-scaled training data, consisting of all subjects except the one currently left out as test patient. Subsequently the input IDIFs of the test subject was scaled by multiplication of the scale factor. The scaled IDIFs were used as input into the trained MLIF model, to generate a predicted scaled MLIF curve. The scale-dependency of the MLIF model was evaluated by comparing the area-under-curve (AUC) of the scaled input IDIFs and AUC of the corresponding scaled output MLIF curves. Scale factors, s, in the range 0.5 to 2 were investigated (s = 0.5, 0.75, 1.0, 1.25, 1.5, 2).

Figure S2A displays the generated MLIF AUC as a function of the scaled IDIF AUCs for all subjects. This graph shows that the model-generated MLIFs scales non-linearly with scaled IDIFs. Furthermore, it can be deduced that the model works well within the range of the training data (scale, s = 1). This can be expected for any data-driven, machine learning-based method. The model breaks for higher scale factors, at IDIFs with an AUC above 2000 kBq·s/ml, where the AUCs are about 1.3 times the values used for training. There is also a tendency for the lowest scale factor data (s = 0.5) to fail at the values furthest from the range of the training data (s = 1). Figure S2A suggest that it is safe to predict MLIF data within the training range, but also for data with amplitudes (or AUC) within a range being extended from the training data range by 25% (s=0.75 to 1.25)

These results confirm that the normalization used as input for the training does not affect the MLIF model's capability to infer the MLIF amplitude from the IDIF.

The reason for the non-linear dependency of the MLIF AUC on IDIF scaling can be understood from Figure S2B. Also here, in the original arterial input data, there is a non-linear dependency, in this case between the IDIF and the measured AIF AUC values. This non-linearity may be hard to appreciate from Figure S2B but becomes more obvious considering that a line-fit has a slope of 1.5 for the range of training data. Furthermore, the y-intercept of the linear fit is >> 0 kBq·s/ml, which is not describing the reality where zero blood input would give zero IDIF from images.

To summarize, this observation suggests that the non-linear relationship observed at different scaling of the IDIF (Figure S2A) is a feature carried over from the original data. Thus, the non-linearity is not a defect by the MLIF-model but arise from the non-linear scaling of the AIF with the scaled IDIFs (Figure S2B). This is in itself an interesting observation, which may arise from the influence of the neighboring tissue to the pixels defining the IDIF. However, further investigation of this effect is outside the scope of the current work.

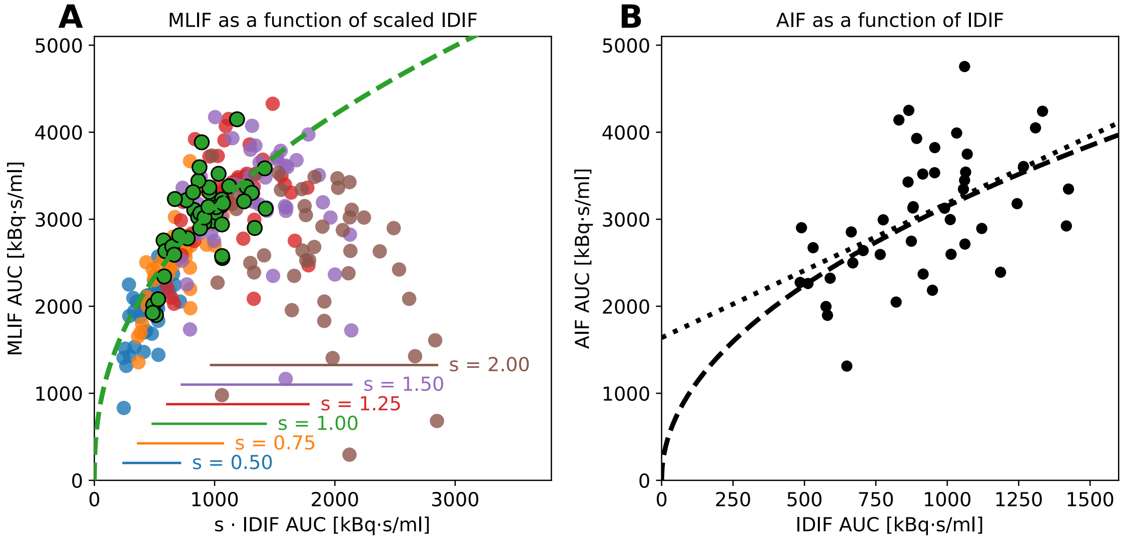

Figure S2. A. Area-under-curve (AUC) of predicted MLIF for different scaled input-IDIF AUCs. IDIF AUC is calculated as the mean AUC of $IDIF_{10}$, $IDIF_{100}$ and $IDIF_{1000}$. Colors indicate different scale factors, s. Green circles display non-scaled values (s = 1). Horizontal bars indicate the AUC range of predicted MLIF AUC values for each scale factor. The graph indicates that the model explains data well within the trained range but breaks down when extending input data too far outside the trained range (s = 1). The curve is a fit to a power function on the non-scaled data, as a visual guide (dashed green line). B. AUC of the measured AIF and IDIF (not scaled) show that the AIF does not scale linearly (linear fit: $y=1.5x+1639$) with IDIF, but rather follows a power fit (dashed line).
